# Gender differences in prevalence of hepatitis C virus infection in Egypt: a systematic review and meta-analysis

**DOI:** 10.1038/s41598-023-29262-z

**Published:** 2023-02-13

**Authors:** Muhammad Abdel-Gawad, Mohamed Nour, Fathiya El-Raey, Hanaa Nagdy, Yahya Almansoury, Mohamed El-Kassas

**Affiliations:** 1 Hepatology, Gastroenterology, and Infectious Diseases Department, Assiut Faculty of Medicine, Al-Azhar University, Assiut, Egypt; 2 Department of Public Health and Community Medicine, Damietta Faculty of Medicine, Al-Azhar University, Damietta, Egypt; 3 Faculty of Public Health and Health Informatics, Umm Al-Qura University, Mecca, Saudi Arabia; 4 Hepatogastroenterology and Infectious Diseases Department, Damietta Faculty of Medicine, Al-Azhar University, Damietta, Egypt; 5 Internal Medicine Department, College of Medicine, Arab Academy for Science and Technology and Maritime Transport, Alexandria, Egypt; 6 Internal Medicine Department, Gastroenterology and Hepatology Division, South Valley University, Qena, Egypt; 7 Endemic Medicine Department, Faculty of Medicine, Helwan University, Ain Helwan, Cairo, 11795 Egypt

**Keywords:** Gastroenterology, Health care, Medical research

## Abstract

Egypt is the country with the highest known hepatitis C virus (HCV) prevalence worldwide. The origin of gender differences in HCV prevalence is not usually well understood. This systematic review and meta-analysis aimed to review and evaluate the gender differences in HCV infection rates amongst Egyptians. Such data would be important to support prevention and control programs aiming to minimize HCV-related morbidity and mortality. PubMed, Scopus, and Web of Science (WOS) were searched for relevant articles published from 1st January 2011 to 13th December 2021, using the search terms (HCV OR “hepatitis C” OR hepacivirus) AND (prevalence OR seroprevalence OR epidemiology OR incidence OR magnitude). At first, retrieved articles were screened, and then relevant data were extracted and analyzed. Descriptive statistics were used for data analysis. Out of 616 studies from databases, only 30 were included after the full-text screening, with 193,621 included participants: 97,597 male and 96,024 female. The overall seroprevalence of HCV antibodies in all included studies was 0.02 (CI − 0.23 to 0.28), with no significant difference between males and females. However, HCV RNA positivity was significantly more prevalent in males than females in adults and the general population (after excluding high-risk groups). In children, no statistically significant differences between males and females were found in the seroprevalence of HCV antibodies nor in the prevalence of PCR positivity. HCV RNA positivity is significantly higher in males than females in adults, while there are no gender differences in children.

## Introduction

Hepatitis C virus (HCV) infection is a significant public health concern and, regrettably, a major cause of liver-related morbidity and mortality that challenges healthcare systems in many countries. Globally, 1.5 (1.3–1.8) million people are newly infected with HCV every year, and 58 (46–76) million people are living with chronic HCV infection, with a global prevalence of 0.8% (0.6–1.0%) in the general population. The highest prevalence in the Eastern Mediterranean Region is 1.6% (1.4–1.8%), 290,000 (230,000–580,000) people die from hepatitis C-related causes every year, and only 21% of people are diagnosed with HCV infection, and 62% of them receive treatment^1^.

Gender is an essential determinant of social outcomes, including health. Research has shown a growing interest in health-related gender differences and raises the question of gender-biased differential response that is relevant in many health fields, including the prevalence, risk factors, clinical features, and treatment of diseases. Still, the epidemiological pattern of HCV infection in research and medical practice requires further knowledge of the potential role of gender differences. Thus, assessing gender-level change in HCV prevalence may help identify population subgroups most likely to suffer an increased infection rate, thus enabling health authorities to plan targeted interventions for these changes^2^.

The origin of gender differences in HCV prevalence is not well understood, and some hypotheses tried to explain this difference. The salience of gender in positioning women at increased risk of exposure to HCV infection has been confirmed by some studies^3–6^, while others support the view that HCV infection appears to be prevalent and progresses more rapidly in males than in females^7–11^. On the other hand, some studies found gender differences are artifactual, with nearly flat rates of HCV infection^12–15^. Also, some studies found a decreased rate of liver cirrhosis and hepatocellular carcinoma (HCC) in females^16^, more progress to hepatic fibrosis in males, and more liability to adverse events of direct-acting antivirals (DAAs) in females^17^.

Compared to men, women are more exposed to syringes, blood, and blood products, especially during pregnancy and labor, and ear piercing, and thus run a higher risk of HCV infection. Biological sex with female predominance has been associated with differences in rates of spontaneous HCV clearance, with a possible role of sex hormones in determining host susceptibility to viral infections^18^. On the other hand, male predominance can be explained by differences in daily life conditions, environmental experiences, and social, cultural, and occupational aspects taken up more frequently by men than women, especially in marginalized groups and slum areas such as IV drug use, circumcision, shared use of toothbrushes or shaving razors, tattooing, wet cupping (Higama), or illegal sexual intercourse, in addition to blood transfusion emergencies^19^. The hormonal hypothesis and other mechanisms have been invoked, such as cellular mosaicism, genes escaping X chromosome inactivation, skewed X chromosome inactivation, and miRNAs encoded on the X chromosome^20^. In addition, gender differences in HCV infection rates might reflect differences in the patterns of gender-specific risky behaviors^21,22^.

Historically, Egypt is one of the world countries with the highest prevalence of HCV infection. Over the past decade, Egypt has continued efforts to achieve HCV control and works towards the common goal, targeted by the WHO, of the elimination of viral hepatitis by 2030. The universal access to treatment with the introduction of DAAs has resulted in a paradigm shift in HCV management and declining mortality. A large Egyptian study showed a marked decrease in mortality in Egypt^23^.

In Egypt, some studies indicated that anti-HCV prevalence in the general population was higher in males than in females (19.67% vs. 9.73%; *p* < 0.001)^10^, (16.1% vs. 13.4%; *p* < 0.001)^9^, and (7.5% vs. 5.3%; *p* < 0.001)^8^. While other studies found more anti-HCV prevalence in women than in men in special situations: (13.4% vs. 7.3%; *p* = 0.045) among family contacts of HCV-positive children^24^, (25.2% vs. 17.6%; *p* = 0.031) in patients with coronary heart disease^5^, and (25.1% vs. 15%; *p* = 0.002) in apparently healthy blood donors^3^.

We aimed to review and evaluate the dominance of gender in HCV infection and whether gender differences in HCV seroprevalence and HCV RNA exist amongst Egyptians that can support prevention and control programs and minimize HCV-related morbidity and mortality.

## Methods

To conduct this meta-analysis, we searched PubMed, Scopus, and Web of Science (WOS) for relevant articles published from 1st January 2011 to 13th December 2021. Our protocol was registered to the International Prospective Register of Systematic Reviews (PROSPERO, CRD42022303921).

### Search strategy

We searched the target databases one by one using the following search terms: (HCV OR “hepatitis C” OR hepacivirus) AND (prevalence OR seroprevalence OR epidemiology OR incidence OR magnitude).

### Eligibility criteria

Any Egyptian observational cross-sectional study containing original information regarding the prevalence of HCV on both males and females published in the English language from 2011 or later till the end of 2021 was included irrespective of the governorate, studied group, and age of participants. Studies that did not specify gender, non-Egyptian studies, studies with a mixed population with no definite data for Egyptians, studies in which prevalence was not the primary concern, editorials, reviews, abstracts, posters, commentaries, and non-human studies were excluded.

### Screening, data extraction, and quality assessment

Title and abstract screening were done by four independent reviewers (**MO, YA, HN, FA**), and a discussion with the research team solved any disagreements. The same four independent reviewers did full-text screening for articles selected in the previous step. Data extraction for finally included studies retrieved from the full-text screening was done independently. Relevant data were extracted to a pre-prepared excel file. Quality assessment was done by two independent reviewers (MA, MA). We used The Joanna Briggs Institute (JBI)^25^ tool for the quality assessment of prevalence studies.

### Data synthesis and analysis

Collected data were analyzed using STATA version 16 (Stata Crop LP). Heterogeneity was assessed using the I^2^ test and classified as high, moderate, and low heterogeneity according to I^2^; more than 75%, 50%, or 25%, respectively. A log odds ratio with a 95% confidence interval (CI) was used with a random effect model to compare males and females. A funnel plot was used to check for publication bias using egger's test (a *p* value less than 0.05 was considered significant) (Supplementary Fig. 1).

## Results

### Overview of included studies

**Figure 1:**
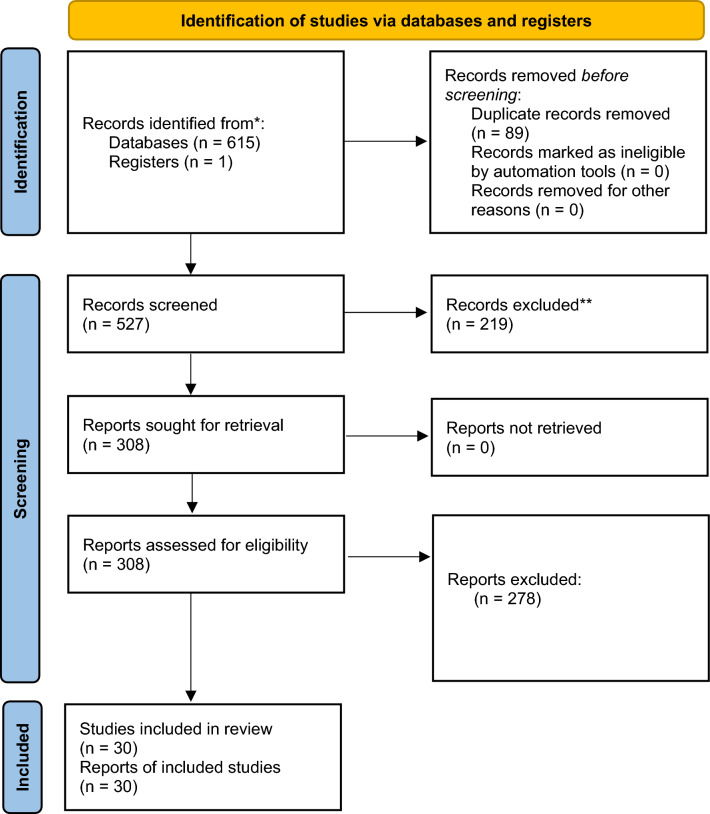
PRISMA 2020 flow diagram for new systematic reviews which included searches of databases and registers only.

Our primary search results yielded 616 studies from databases; 89 duplicated studies were excluded. By title and abstract screening, 308 studies were selected and entered the full-text screening. Only 30 studies^3–5,8–10,12–15,24,26–44^ were finally included in our meta-analysis after full-text screening (Fig. 1) with 193,621 included participants: 97,597 male and 96,024 female. Studies ranged from modest to high quality. Characteristics of the included studies are detailed in Table 1.

**Table 1:** Characteristics of finally included studies.

Authors	Year	Region	Study type	Used test	Studied population	Age	Age group	Participants	Males	Females	Overall HCV Antibodies	Overall PCR positivity	Males HCV Antibodies	Males PCR positivity	Females HCV Antibodies	Females PCR positivity
El-Faramawy 2012	2012	Qena Governorate	Cross sectional	Second generation ELISA	Multitransfused children	8.29 ± 3.16	Children	100	68	32	45		32		13	
El Garf 2012	2012	Cairo University Hospital	Cross sectional	Third generation ELISA	Hospitalized patients	32.7 ± 13.1 (12–69)	Mixed	157	24	133	29	15	4		25	
Awadalla 2011	2011	Cairo University Hospital	Cross sectional	ELISA	Blood donors	18–60	Adults	1000	825	175	168		124		44	
Abdelwahab 2011	2011	National Liver Institute, Menufia	Cross sectional	Third generation ELISA	Health Care Workers	31.5 ± 9.4	Adults	842	384	458	140	101	92		48	
Abd Elrazek 2014	2014	Several medical canters in urban and rural areas across Egypt	Cross sectional	ELISA and PCR	General population	17–58	Adults	6660	3836	2824	1580	1018		627		391
Talaat 2019	2019	Abbasia, Alexandria, Helwan, Menouf, and Aswan	Cross sectional	ELISA	Suspected hepatitis	(1–90) mean 13.9	Mixed	9321	5471	3850	252		191		61	
Soliman 2019	2019	Luxor	Cross sectional	Third generation ELISA	General population	Mean 43.6 years, Median 43 years, and Range 22 years,	Adults	67,042	31,965	35,077	9701		6288		3413	
Sherief 2021	2021	Menoufia and Sharkia	Cross sectional	ELISA and PCR	Multitransfused children	9.9 ± 5.1	Children	477	262	215	70	69	38	37	32	32
Sherief 1 2019	2019	Sharkia	Cross sectional	ELISA and PCR	Relatives of oncology patients	18,537	Mixed	450	204	246	48	57	15		33	
Dahab 2019	2019	Magrabi eye hospital, Cairo	Cross sectional	rapid chromatography immunoassay	Ocular surgery patients	50.85 ± 19.77	Adults	3067	1592	1475	380	380	215		166	
Anwar 2021	2021	Ain Shams University Hospitals	Cross sectional	ELISA	Hospitalized patients	54.35 ± 14.46	Adults	500	288	212	99		67		32	
Anwar1 2021	2021	Health-care workers of Ain Shams University hospitals	Cross sectional	ELISA	Health Care Workers		Adults	50	17	33	4		1		3	
Ahmed 2020	2020	Qena	Cross sectional	ELISA	Blood donors		Adults	11,604	10,232	1372	370		326		326	
Abo-Amer 2018	2018	Lower Egypt, n = 47,344 (97%) Male, n = 21,365 Female, n = 25,979 Upper Egypt, n = 1448 (3%) Male, n = 6 Female, n = 1442	Cross sectional	ELISA and PCR	University students	18 ± 0.056	Adults	48,792	21,371	27,421	498		194	147	304	208
Mohlman 2015	2015	Birthplace Urban 16.7% Rural 37.5% Residence at time of interview Urban 22.2 Rural 36.9%	Cross sectional	ELISA and PCR	Control group of hepatocellular Carcinoma patients	> 17	Adults	1764	1094	670	525	404	352		169	
Abdelmoemen 2018	2018	Tanta University Hospitals	Cross sectional	HCV RNA viral load in the plasma and PBMCs by standardized quantitative real-time PCR	Haemodialysis patients	44.5 ± 13.8	Adults	62	35	27		3		2		1
Abd El Salam 2016	2016	Zagazig University Hospitals, Sharkia	Cross sectional	HCV Antibodies	Coronary artery disease patients	53.37 ± 8.36	Adults	344	375	206	118		66		52	
Mansour 2012	2012	Dakahlia, Mansoura University Children’s Hospital		HCV Antibodies	Multitransfused children	13 (11 month-19 year)	Children	200	111	89	81		42		39	
Esmat 2016	2016	Upper Egypt and Lower Egypt undergraduate students at the Cairo University Hospital		Serum HCV Antibodies and quantification of HCV load in serum	University students	18.1 ± 0.7	Adults	3000	1340	1660	137	43	51	29	86	14
Emam 2015	2015	Sharkia		HCV antibodies	Elderly population	64.37 ± 4.74	Adults	214	115	99	60		32		28	
Elhendawy 2020	2020	Gharbia, Basyoun, village of Nagreej	Prospective cohort	PCR	General population	18–60	Adults	2048	930	1118	542	505		282		223
El-Ghitany 2019	2019	21 Governorates: Alexandria, Asyut, Beheira, Beni suef, Cairo, Dakahlia, Damietta, Faiyum, Gharbia, Giza, Ismailia, Kafr El-Sheikh, Luxor, Marsa matruh, Menoufeya, Minya, Port Said, Qalubeya, Sharqeya, Sohag, Suez	Cross sectional	Serum HCV Antibodies and quantification of HCV RNA load in serum	General population	38.95 ± 13.3 (14–90)	Mixed	12,169	6170	5999	1795	1070	993		802	
El Garf 2013	2013	Cairo University Hospitals	Cohort	Serum HCV Antibodies and quantification of HCV RNA load in serum	SLE patients	14–63 (26.5)	Mixed	98	11	87	20	8	2		18	
Edris 2014	2014	Damietta	Cross sectional	HCV antibodies	General population	33.9 ± 15.6	Mixed	2977	1621	1356	278		160		118	
El Feki 2013	2013	Beni-Suef	Cross sectional	Serum HCV Antibodies and quantification of HCV RNA load in serum	General population	15–70	Adults	400	235	165	144	136	84		60	
El Batae 2018	2018	Kafr El Sheikh University	Cross Sectional	PCR	University students	18.6 ± 0.39	Adults	9049	4233	4816	25	24		13		11
Barakat and El-Bashir 2011	2011		representative sample	Serum HCV Antibodies and quantification of HCV RNA load in serum	General population	6–15 years	Children	500	254	246	29	22	18		11	
Abd El-Wahab 2016	2016	Alexandria	Cross sectional	Serum HCV Antibodies and quantification of HCV RNA load in serum	School children	6 and 15 years	Children	500	284	216	14		8		6	
Ibrahim 2016	2016	Electricity generating company in Mansoura	Cross sectional	HCV Antibodies	General population	21–62 (40.61)	Adults	258	216	42	38		36		2	
MOH Survey 2015	2015	All over Egypt	Cross sectional	ELISA and PCR	General population	1–59 years	Mixed	27,549	13,068	14,481	1735	1212	980	692	767	521
						1–14 years	Children	10,878	5606	5272	43	21	39	11	10	5
						15–59 years	Adults	16,671	7462	9209	1667	1166	925	664	745	506

### The overall gender differences in the seroprevalence of HCV antibodies (all included studies)

**Figure 2:**
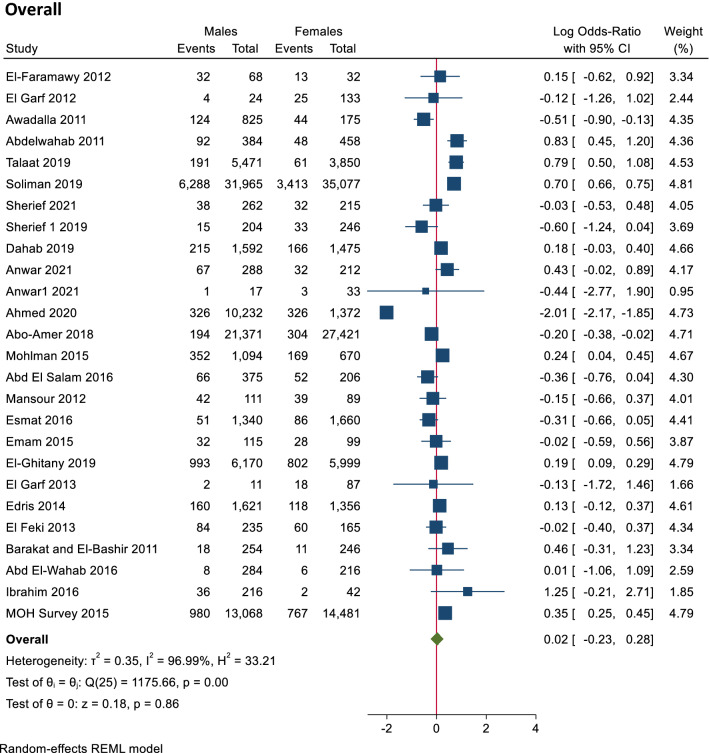
Forest plot of seroprevalence of HCV antibodies in males and females. There is no significant difference in seroprevalence of HCV antibodies between males and females.

The seroprevalence of HCV antibodies among the Egyptian population using serum antibodies test by ELISA, based on the results of the random-effects method, there were no significant differences between male and female seroprevalence of HCV. The overall seroprevalence was 0.02 (log 95% CI − 0.23, 0.28), and the heterogeneity was high (T^2^ = 0.35, I^2^ = 96.99, H^2^ = 33.21, *p* value = 0.86) as shown in Fig. 2.

### Gender differences in the prevalence of HCV PCR positivity

**Figure 3:**
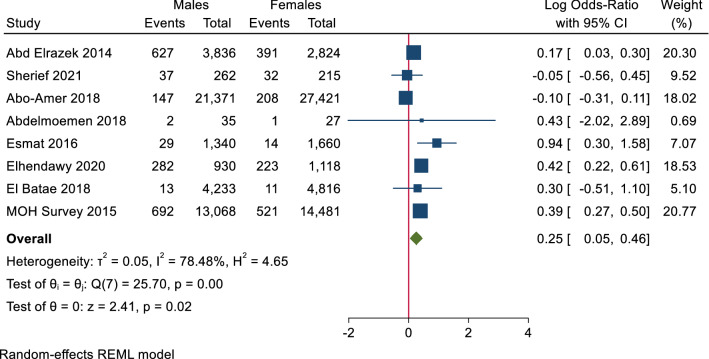
Forest plot of prevalence of HCV RNA in males and females. There is statistically significant increase in prevalence of HCV RNA positivity in males than females.

Eight studies used PCR to measure the prevalence of HCV; based on the results of the random-effects method of these studies, there was a statistically significant increase in the prevalence of HCV RNA positivity in males than females (0.25, log 95% CI 0.04–0.46) and the heterogeneity was high (T^2^ = 0.05, I^2^ = 78.48, H^2^ = 4.65, *p* value = 0.02) as shown in Fig. 3.

### Subgroup analysis of gender differences

#### Children versus adults

**Figure 4:**
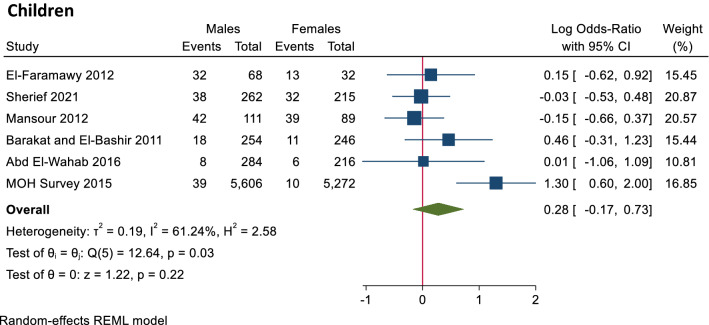
Forest plot of seroprevalence of HCV antibodies in males and females in children. There is no significant difference between males and females in HCV antibodies prevalence.

**Figure 5:**
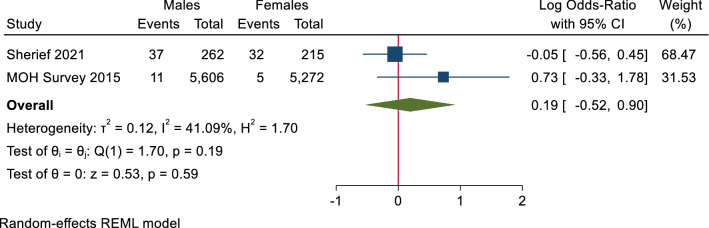
Forest plot of prevalence of HCV RNA in males and females in children. There is no significant difference between males and females in HCV RNA prevalence in children.

**In children,** there were no statistically significant differences in the seroprevalence of HCV antibodies or prevalence of PCR positivity (Figs. 4, 5).

**Figure 6:**
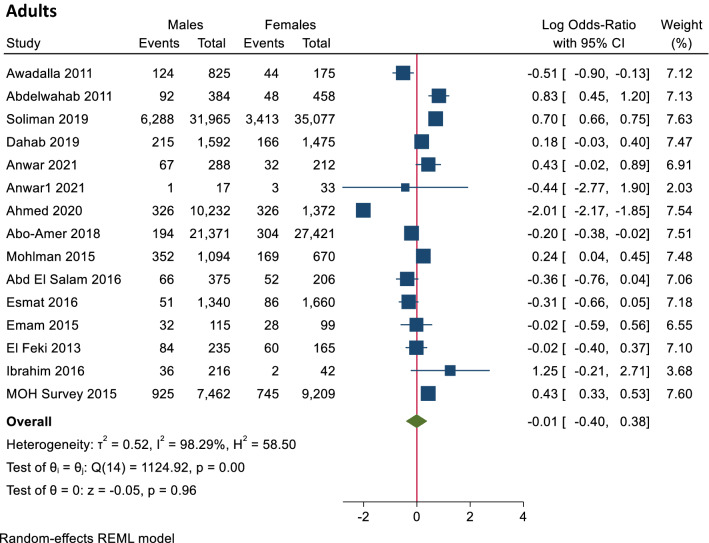
Forest plot of seroprevalence of HCV antibodies in males and females in adults. There is no significant difference in seroprevalence of HCV antibodies between males and females in adults.

**Figure 7:**
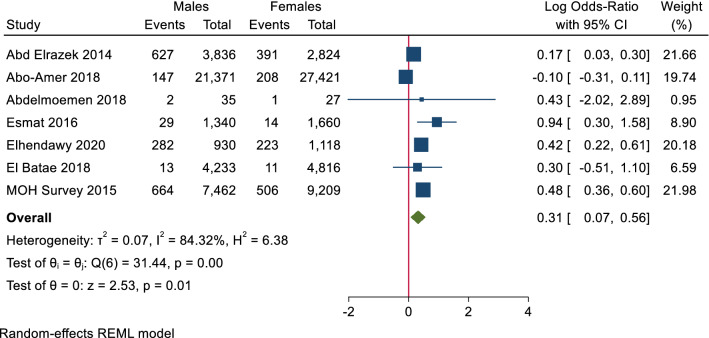
Forest plot of prevalence of HCV RNA in males and females in adults. There is statistically significant increase in prevalence of HCV RNA in males than females in adults.

In adults, there were no significant differences in the seroprevalence of HCV antibodies between males and females. At the same time, HCV PCR testing showed a significant increase in the male prevalence of HCV PCR positivity to females (0.31, log 95% CI 0.07–0.56), and the heterogeneity was high (T^2^ = 0.07, I^2^ = 84.32, H^2^ = 6.38, *p* value = 0.01) (Figs. 6, 7).

#### General population

By studying gender prevalence differences in studies conducted on the general population (with no risk factors) after excluding studies on high-risk groups, males showed a significantly increased prevalence of both HCV antibodies and HCV PCR positivity (Supplementary Figs. 2, 3).

## Discussion

Gender-based differences vary by country and region. This study presents a comprehensive update on HCV infection gender differences in Egypt. Despite the historic large-scale epidemic in Egypt, HCV antibody incidence and prevalence appear to decline rapidly, consistent with a contracting epidemic. In 2006, the national treatment strategy for the control of HCV infection in Egypt was established in response to the magnitude of the HCV problem and the disease burden in Egypt^45^.

Egypt is the country with the highest HCV prevalence worldwide. Six percent of individuals aged 1–59 years had a positive result on the hepatitis C antibody test, and 4% were found to have an active infection. The prevalence of hepatitis C was higher among men than women in most age groups^8^. This finding agreed with the results of this meta-analysis in all studies conducted on the general population (26 studies).

There is limited published data on gender-based differences in children^46^. The worldwide pooled seroprevalence of HCV in children in these studies was low, < 1%^47^. In 2015, the Egyptian Demographic and Health Surveys (EDHS) tested 10,044 children (5154 male and 4890 female) to estimate HCV prevalence in those aged 1–14. The results showed that HCV prevalence in the group aged < 15 years was 0.4%. HCV antibody seroprevalence was 0.7% with viremia of 0.2% in male children, while HCV antibody was seroprevalent in 0.2% with the presence of viremia in 0.1% of female children^10^. The current meta-analysis shows no significant differences between male and female children in HCV antibody prevalence or viremia.

The EDHS reported that seroprevalence of HCV antibodies was 14.7% among the adult population aged 15–59 years at 14.7% with a national viremic prevalence of 9.7%, which was higher in males than in females in all studied age groups in 2008^48^. Similarly, the results of this meta-analysis showed that the prevalence of HCV viremia was significantly higher in males than females in adults. This difference may be attributed to males being more affected by schistosomiasis disease burden and hence were the main target of the parenteral antischistosomal therapy (PAT) campaign^49^ with high risk for parenteral virus transmission, including HCV. Also, the lifestyle of males makes them more exposed to various risk factors for HCV transmission^50^.

In 2015, there was significantly lower HCV prevalence in those aged 15–19 years compared to the 2008 data (14%), and this points to a significant decrease in new infections in the age groups 15–19 years^51^. Total HCV seroprevalence in the age groups 15–59 years was 10%, with a viremic prevalence of 7%. HCV seroprevalence in female adults was 8.1%, with viremia of 5.5%, while HCV seroprevalence in male adults was 12.4%, with a viremic prevalence of 9.8%^8^.

### Study limitations

There were not enough data from the included studies to determine the sources of heterogeneity. It may be due to differences in populations, regions, geographical locations, seasons, settings (rural or urban), and used screening methods. Unmeasured covariates (such as population characteristics, presence of comorbidities, HIV status, etc.) could have contributed to variability in outcome estimates. Gender differences among high-risk populations (e.g., healthcare workers, drug users, incarcerated populations, people living with HIV, etc.) were not considered in this study to avoid overestimating the problem.

## Conclusion

HCV RNA positivity is significantly higher in males than females in adults, while there are no gender differences in children.

## Supplementary Information

Supplementary Figures.

## Data Availability

The data that support the findings of this study are available from the corresponding author upon reasonable request.
