# Supplementary material for: Gender differences in prevalence of hepatitis C virus infection in Egypt: a systematic review and meta-analysis
Source: Sci Rep. 2023 Feb 13;13:2499. doi: 10.1038/s41598-023-29262-z (PMC9925441; doi:10.1038/s41598-023-29262-z)
Supplement: Supplementary file 1 — Supplementary Figures. [file 41598_2023_29262_MOESM1_ESM.docx]

**Supplementary figures 1-3**

**Supplementary figure 1**: Funnel plot for publication bias

1. **Prevalence in studies conducted on the general population as a subgroup analysis.**

**General population (after exclusion of all studies done on specific groups)**

**Supplementary Figure 2: Forest plot of seroprevalence of HCV antibodies in males and females in the general population**

There is statistically significant increase in seroprevalence of HCV antibodies in males than females in the general population.

**Supplementary Figure 3: Forest plot of prevalence of HCV RNA in males and females in the general population**

There is statistically significant increase in prevalence of HCV RNA in males than females in the general population.
